# Visual Occipito-Temporal N1 Sensitivity to Digits Across Elementary School

**DOI:** 10.3389/fnhum.2022.887413

**Published:** 2022-07-26

**Authors:** Gorka Fraga-González, Sarah V. Di Pietro, Georgette Pleisch, Susanne Walitza, Daniel Brandeis, Iliana I. Karipidis, Silvia Brem

**Affiliations:** ^1^Department of Child and Adolescent Psychiatry and Psychotherapy, Psychiatric University Hospital Zurich, University of Zurich, Zurich, Switzerland; ^2^Neuroscience Center Zurich, University of Zurich and ETH Zurich, Zurich, Switzerland; ^3^MR-Center, Psychiatric University Hospital Zurich, University of Zurich, Zurich, Switzerland; ^4^Center for Interdisciplinary Brain Sciences Research, Stanford University School of Medicine, Stanford, CA, United States

**Keywords:** ERP, visual N1, occipito-temporal cortex, number processing, development, numeracy, arithmetic skills

## Abstract

Number processing abilities are important for academic and personal development. The course of initial specialization of ventral occipito-temporal cortex (vOTC) sensitivity to visual number processing is crucial for the acquisition of numeric and arithmetic skills. We examined the visual N1, the electrophysiological correlate of vOTC activation across five time points in kindergarten (T1, mean age 6.60 years), middle and end of first grade (T2, 7.38 years; T3, 7.68 years), second grade (T4, 8.28 years), and fifth grade (T5, 11.40 years). A combination of cross-sectional and longitudinal EEG data of a total of 62 children (35 female) at varying familial risk for dyslexia were available to form groups of 23, 22, 27, 27, and 42 participants for each of the five time points. The children performed a target detection task which included visual presentation of single digits (DIG), false fonts (FF), and letters (LET) to derive measures for coarse (DIG vs. FF) and fine (DIG vs. LET) digit sensitive processing across development. The N1 amplitude analyses indicated coarse and fine sensitivity characterized by a stronger N1 to digits than false fonts across all five time points, and stronger N1 to digits than letters at all but the second (T2) time point. In addition, lower arithmetic skills were associated with stronger coarse N1 digit sensitivity over the left hemisphere in second grade (T4), possibly reflecting allocation of more attentional resources or stronger reliance on the verbal system in children with poorer arithmetic skills. To summarize, our results show persistent visual N1 sensitivity to digits that is already present early on in pre-school and remains stable until fifth grade. This pattern of digit sensitivity development clearly differs from the relatively sharp rise and fall of the visual N1 sensitivity to words or letters between kindergarten and middle of elementary school and suggests unique developmental trajectories for visual processing of written characters that are relevant to numeracy and literacy.

## Introduction

Digits are symbols culturally associated with a representation of magnitude or quantity. Familiarization with numbers begins early in life, initializing basic processes that may have important consequences to the development of arithmetic and other academic skills (Bartelet et al., 2014; Lau et al., 2021). A frequently used model to describe number processing is the triple-code model (Dehaene, 1992; Dehaene et al., 2003), which proposes a set of separate systems for the representation of visual, verbal and quantity information. Each of these systems is supposed to rely on the specialization of sensory and associative brain areas (Dehaene, 2011), in a similar way as literacy leads to the development of brain networks for reading (Dehaene and Cohen, 2007). Understanding the development of specialized brain networks for digit and number processing could help characterize numeracy-related learning difficulties, such as dyscalculia (Butterworth et al., 2011).

The hierarchical organization of the human visual cortex allows for functional specialization of category-selective brain regions in ventral occipito-temporal cortex. In studies using electroencephalography (EEG), neural responses in vOTC can be captured with the visual N1, an event-related potential peaking around 130–230 ms over occipito-temporal electrodes (Nobre et al., 1994). The amplitude and latency of the visual N1 is able to capture category-selective visual responses to faces, bodies, and objects (Bentin et al., 1996; Thierry et al., 2006), as well as to words and numerals (Bentin et al., 1999). A study using intracranial electrophysiological recordings identified a region in the right inferior temporal gyrus showing preferential activation, i.e., stronger responses, to visual symbols denoting numerals compared to letters and false fonts (Shum et al., 2013). Of note, numerals in Shum et al. (2013) engaged different neuronal populations than words and letters in the right hemisphere. In the left hemisphere, even though there was a less dense electrode coverage, responses to digits were of similar strength to those of false fonts or letters, and comparable to the responses of digits in the right hemisphere. The category-selective response that Shum et al. (2013) demonstrated in the right inferior temporal gyrus introduced the notion of a right-lateralized number form area, analogous to other category-selective vOTC subregions, such as the visual word form area (Dehaene and Cohen, 2011) and the letter form area (Thesen et al., 2012) in the left fusiform gyrus.

A recent meta-analysis of neuroimaging studies supports the existence of a “number form area” specialized for processing Arabic numerals in the right inferior temporal gyrus (Yeo et al., 2017). Even though several studies using non-invasive neuroimaging methods have described number-specific subregions in the visual cortex, their detection is not always consistent. Inconsistent findings to detect a number form area have been attributed to differences in task demands and to methodological limitations of fMRI associated with signal dropouts in the inferior temporal gyrus (Yeo et al., 2017). For example, some meta-analyses suggest high convergence of functional activations to numbers in occipital areas (Arsalidou and Taylor, 2011; Sokolowski et al., 2017) but they do not differentiate between symbolic (e.g., Arabic) and non-symbolic (e.g., dots) stimuli. It has also been suggested that digits and letter strings may be represented as different patterns of activity in the visual cortex rather than recruiting separate regions (Peters et al., 2015). Nevertheless, there is compelling evidence suggesting that bilateral parts of vOTC show number-selective responses compared to letters, false fonts, and objects (Grotheer et al., 2016). In summary, neuroimaging evidence supports the dissociation between numbers and letters, and that some parts of the vOTC respond stronger to numerals and number strings compared to other printed visual categories like words or formulas (e.g., Amalric and Dehaene, 2016). This dissociation, could explain why alexia patients with severe deficits in word and letter reading can have less impaired or intact abilities to read numbers [see review in Starrfelt and Behrmann (2011)].

There is currently limited evidence on how visual specialization to numbers develops in childhood, during the acquisition of numeric and arithmetic skills. Some fMRI studies compared children and adults using explicit tasks involving number comparisons and simple operations (Kucian et al., 2008; Skagenholt et al., 2021). One of them suggested weaker activation for children in third and sixth grades vs. adults in occipital areas during non-symbolic magnitude comparisons, in the inferior frontal gyrus when performing (symbolic) exact calculations and in the intraparietal sulcus across tasks (Kucian et al., 2008). Another study showed weaker activations in left-perisylvian language areas in 10–12-year-old children compared to adults during a verbal number comparison task, but not during an Arabic digit comparison task (Skagenholt et al., 2021). In addition, an fMRI study examined longitudinally 8–11-year-old children with and without developmental dyscalculia, using a numerical order judgment task and two time points during a period of 4 years (McCaskey et al., 2018). The study found an increase in activation over time in frontal and parietal regions in children with dyscalculia, but a stable pattern of activations in controls. Stronger occipito-temporal activations in children with dyscalculia were detected only when using a less strict significance threshold. These studies provide insights regarding brain networks for arithmetic, ordinal and quantity processing, but did not focus on the basic visual processing of numbers.

The current study uses EEG to investigate the development of visual specialization to single digits in children from pre-school through elementary school, covering five time points and combining cross-sectional and longitudinal data. A few previous studies addressed visual processing of digits using EEG. Lochy and Schiltz (2019) investigated discrimination between digits and letters in 6-year-old children with a fast (<170 ms per stimulus) periodic visual stimulation approach. The frequency-tagging approach in this study found right-lateralized responses in posterior-occipital electrodes to digits embedded as deviants in streams of letters, and a trend for left-lateralized responses to letters embedded in digit streams, which is in line with previous fMRI data on adults (Park et al., 2012). Whether such differential processing of letters and digits is already present in even younger children is uncertain because a previous fMRI study on 4 year-old children, did not find differences between processing letters and digits, although both stimulus categories elicited greater responses compared to objects (Cantlon et al., 2011).

Another EEG study used a similar fast periodic visual stimulation in adults to investigate magnitude and parity processing of Arabic digits (Guillaume et al., 2020). Small numbers were embedded as deviants in streams of large numbers to study magnitude processing and odd digits in even digit streams to study parity (the reverse contrasts were also presented). Deviants in both magnitude and parity elicited significant discrimination responses at the corresponding frequencies over right occipito-parietal electrode sites. These responses suggest processing of semantic properties even in implicit tasks with fast presentation rates, and their topographies are consistent with the suggested involvement of parietal regions in holding semantic number representations (Dehaene et al., 2003). Several studies have also investigated semantic number processing by examining ERPs in numerical Stroop tasks, in which a decision on perceptual features of stimuli is facilitated or interrupted by automatic access to semantic information (e.g., deciding if two digits are numerically or physically larger). In one of these studies 5–6-year-old children showed occipito-temporal sensitivity to distance in numerosity/size and frontal and parietal responses to conflict in this task (Ben-Shalom et al., 2013), in line with a previous report using a similar paradigm in adults (Szucs and Soltész, 2008). Another ERP study showed right parietal responses to numerical distance in 9–11-year-old children and adults (Szucs et al., 2007). Finally, two other studies found similar timing of ERPs associated with automatic access to numerical representation in 6–8-year-old children and adults (Soltész et al., 2011a,b). Altogether, this set of studies supports that automatic processing of semantic features of digits starts at an early age and that it modulates occipito-temporal and parietal responses. Most of the paradigms used by these studies, however, tackled several cognitive processes such as performance monitoring and inhibition of relevant information, in addition to basic visual number processing.

In the current study we use an ERP approach to examine the visual N1 as the electrophysiological correlate of vOTC activity during a simple target detection task in which single characters are visually presented. Similarly to Lochy and Schiltz (2019), we were interested in brain responses to single digits in contrast to other relevant (letters) and irrelevant (false fonts) characters and how these responses develop across the first years at school. Here, we also use an implicit task that does not require to actively process the stimuli, but instead of focusing on responses to deviants inserted in rapid streams of standard stimuli, we compare the widely studied N1 ERP response to stimuli from different categories (i.e, digit, letter, false font) presented in a target detection task with slower presentation rates. The N1 is an early ERP component that peaks around 170 ms after stimulus presentation and is detected over posterior and bilateral occipito-temporal scalp electrodes. Besides general visual expertise (Tanaka and Curran, 2001), the amplitude of the N1 component has been associated with visual letter and word processing in multiple studies of reading and dyslexia (e.g., Maurer et al., 2006; Brem et al., 2013; Fraga-González et al., 2014; Araújo et al., 2015).

With a similar focus to that of the current study, Park et al. (2017) examined N1 responses to strings of numbers, letters, and false fonts in a cross-sectional design with 7-, 10-, 15-year-olds, and young adults performing a target detection task. The results suggested a different trajectory for the fine contrast of letters vs. numbers, compared to the coarse contrast of familiar vs. unfamiliar stimuli (i.e., letters or numbers vs. false fonts). N1 amplitudes over the left and right hemispheres did not discriminate between letters and numbers in the younger 7-year-old group, but became stronger to letters than to numbers over bilateral occipito-temporal scalp electrodes in the 10-year-old children and finally approached adult-like topographies in adolescents with a left-lateralized N1 to letters and a right-lateralized N1 for digits. These rather late lateralization effects somewhat contrast the reported right-lateralized responses for digits embedded in letters found in 6-year-old children by Lochy and Schiltz (2019), although this contradiction is possibly a result of differences in the paradigms, task demands and contrasts. Regarding the coarser discrimination, the two younger groups showed stronger responses for the familiar symbols (digits and letters vs. false fonts) but this pattern changed in the 15-year-old and adult groups, who showed stronger amplitudes for false fonts, in line with a previous report in adults from the same lab (Park et al., 2014).

The study from Park et al. (2017) provides an important reference to understand the development of visual responses to letters and numbers. However, their study design did not cover the initial period when letter and number knowledge, as well as arithmetic and reading skills emerge and rapidly change. The current study combines cross-sectional and longitudinal data to clarify the trajectory of visual N1 specialization to numbers across five time points from preschool to fifth grade and also examines how arithmetic skills may influence this development. We use a similar passive viewing, target-detection paradigm as in Park et al. (2017) involving simpler, single-character stimuli and focus on children only, with measurements starting at preschool. Our task and the focus on the N1 ERP is motivated by the previous extensive literature on the N1 as a proxy for early visual specialization, whereas the stimuli are kept simple to minimize attentional and cognitive load during performance. Our study partially overlaps with the study of Lochy and Schiltz (2019), in that we also presented preschool children with single characters (digits, letters). This allows a comparison of our ERP findings in preschool children to those of the frequency-tagging approach employed by Lochy and Schiltz (2019), and also provides new insights into the further development of coarse and fine specialization for character processing with age and increasing math expertise.

In a previous publication we showed emerging letter sensitivity, i.e., stronger N1 responses to letters than to false fonts in first grade, when children attain advanced letter knowledge, but not in pre-school when letter knowledge is low, or in second and fifth grade when letter-speech sound knowledge is already highly automatized. Thus, an expertise-dependent inverted U-shaped development of letter sensitivity was suggested and interpreted within the predictive coding framework of vOTC specialization (Price and Devlin, 2011). This framework postulates that efficient visual processing is facilitated by top-down predictions, which emerge in early learning stages. This emergence is followed by strong prediction error signals indicating the mismatch between predicted and sensory inputs, which is used to optimize predictions (Friston, 2010). The combination of increased prediction signaling between higher and lower order visual areas and high prediction error signals at this stage of intensive learning explains the peak in neural responses in vOTC. As learning progresses and expertise is attained, predictions become better and prediction error signals decline, resulting in reduced activations. Alternatively, the inverted U trajectory can also be explained by principles of neural plasticity based on *expansion* (growth in number of neurons or connections involved), *selection* of most efficient circuits and subsequent *renormalization* when unnecessary circuits are pruned away (Wenger et al., 2017). These views on neural specialization and skill learning may also apply to learning numbers and arithmetic skills. The present study adds to the previous analysis of N1 specialization to letters by focusing on digit processing and arithmetic skills from kindergarten to elementary school.

The current study focuses on N1 amplitude differences to single digits compared to false fonts as a proxy for coarse vOTC sensitivity to digits as well as on fine sensitivity of visual N1 responses to digits compared to letters. We aim to clarify the trajectory of coarse and fine sensitivity to digits across five time points from kindergarten through elementary school, covering the early and possibly most crucial period in learning basic number processing skills. In contrast to the onset of the N1 letter sensitivity development (Fraga-González et al., 2021) with reading acquisition upon school enrollment (grade 1), we expected digit sensitivity (Lochy and Schiltz, 2019) of the N1 to emerge earlier and therefore to be already present at kindergarten age, when most children have basic knowledge of numbers. Similar to single letter processing, we expected an expertise-dependent change in the N1 digit sensitivity from kindergarten to fifth grade, reflecting the major learning milestones for arithmetics in this period. Unlike letters, we hypothesized that coarse and fine visual N1 sensitivity to digits would be detected bilaterally over occipito-temporal electrodes in kindergarten, i.e., before the emergence of visual letter sensitivity. Later on, as soon as reading skills emerge, fine sensitivity to digits is expected to become more lateralized toward the right hemisphere (Park et al., 2017). However, we should note that no significant lateralization effects were found for processing single-letter characters in our previous analysis (Fraga-González et al., 2021).

The current analysis includes cross-sectional as well as longitudinal data that was collected as part of a larger project focused on literacy acquisition. To achieve variability in reading and math abilities at school age, children with a broad range of familial risk for reading impairments were recruited. Given the frequent comorbidity of developmental dyslexia and dyscalculia (Butterworth et al., 2011; Peng et al., 2020) and the previously reported association of visual word sensitivity to reading skills, we were interested to additionally investigate how visual number sensitivity is related to arithmetic and reading skills in children. For completeness, we also report coarse letter sensitivity (letter vs. false font) which was originally part of our previous work that focused on the development of visual letter sensitivity during literacy acquisition in a largely overlapping sample (Fraga-González et al., 2021). These developmental analyses are followed by an examination of the associations between N1 sensitivity to numbers and cognitive performance, with a special emphasis on arithmetic skills. The overall goal of this study is to understand how basic number processing is reflected in the specialization of the visual N1 and whether individual differences in visual processing of numerals are related to mathematical achievement.

## Materials and Methods

### Participants

The current study combines longitudinal and cross-sectional data from a large sample of German-speaking children who participated in a large project with simultaneous EEG/fMRI sessions, behavioral tests and a grapheme-phoneme intervention training (Karipidis et al., 2017, 2018; Pleisch et al., 2019; Mehringer et al., 2020; Wang et al., 2020; Fraga-González et al., 2021). Here, we focus on data from kindergarten (T1), middle of first grade (T2), end of first grade (T3), middle of second grade (T4), and middle of fifth grade (T5) of elementary school. Only data from participants meeting EEG data quality criteria in at least one time point were analyzed (see section “Event-Related Potential Analysis”). Altogether, 62 participants fulfilled these criteria (35 female). Of these 62 participants, data of 27 children were available at only one time point, data of nine children were available at two time points, data of 10 children were available at three time points, data of 10 children were available at four time points, and data of six children were available at all five time points. An overview of the number of time points for each participant is presented in Supplementary Appendix Figure A1. The number of participants available for each time point were 23, 22, 27, 27, and 42, for T1, T2, T3, T4, and T5, respectively. Based on previous studies (Park et al., 2017; Guillaume et al., 2020; Fraga-González et al., 2021) the current group sizes allow to detect visual N1 effects in our main analyses, although they may be limited for the correlational analysis in some of the time points (see section “Discussion”). Demographic information of the overall sample is presented in Table 1 and the demographic characteristics of the group available at each time point are presented in Table 2. Familial risk for developmental dyslexia was estimated with the Adult Reading History Questionnaire (ARHQ; Lefly and Pennington, 2000). Individual risk scores were defined as the highest parental ARHQ value (Table 1). All participants had non-verbal IQ scores >80, normal or corrected to normal visual acuity, and no neurological or cognitive impairments. Two participants had a diagnosis of ADHD (medication was interrupted 48 h before test sessions), two participants reported having a sibling with reading impairments and one participant had adequate speech abilities during our study but a history of delayed speech development earlier in childhood. All children gave oral assent and parents gave written informed consent. The children received vouchers and presents as compensation for their participation. The project was approved by the local ethics committee of the Canton of Zurich and neighboring Cantons in Switzerland.

**TABLE 1:** Sample characteristics.

	M (SD) [min, max]
*N*	62
Sex ratio (male:female)	27:35
Handedness (right:left)	56:6
Non-verbal IQ^a^	102.46 (8.80) [80, 121]
ARHQ^b^	0.49 (0.16) [0.15, 0.80]

*^a^IQ was estimated from RIAS at T5 in 45 participants, from CFT at T4 in 16 participants, and from HAWIK at T1 in 1 participant.*

*^b^Familial risk level was low in 7 children (11.29%, ARHQ <0.3), moderate in 15 children (24.19%, ARHQ range 0.3–0.4), and high in 40 children (64.51%, ARHQ >0.4).*

**TABLE 2:** Descriptive statistics showing sample characteristics and cognitive assessment scores at each time point.

	T1	T2	T3	T4	T5
	(*N* = 23)	(*N* = 22)	(*N* = 27)	(*N* = 27)	(*N* = 42)
	*M (SD)*	*M (SD)*	*M (SD)*	*M (SD)*	*M (SD)*
Age	6.60 (0.52)	7.38 (0.29)	7.68 (0.30)	8.28 (0.49)	11.40 (0.41)
Sex (female:male)	11:12	16:6	18:9	17:10	27:15
Handedness (left:right)	22:1	21:1	24:3	24:3	38:4
Number-knowledge	14.78 (3.78)	18.36 (2.48)	18.93 (2.40)	20.74 (1.16)	–
**HRT-Arithmetic skills raw**					
Addition	–	–	–	16.15 (3.73)	28.00 (5.15)
Subtraction	–	–	–	14.78 (4.07)	27.43 (5.64)
Completion	–	–	–	8.19 (3.31)	16.24 (5.88)
Comparison	–	–	–	15.22 (4.52)	29.23 (5.79)
Multiplication	–	–	–	8.67 (3.50)	24.17 (6.48)
Division	–	–	–	–	24.22 (7.71)
Speeded writing	–	–	–	19.63 (4.33)	30.10 (5.45)
**HRT-Arithmetic skills (PR)**					
Addition	–	–	–	36.85 (24.47)	39.05 (31.15)
Subtraction	–	–	–	41.96 (24.89)	46.91 (27.87)
Completion	–	–	–	53.56 (26.68)	48.57 (30.17)
Comparison	–	–	–	55.15 (24.46)	61.51 (27.73)
Multiplication	–	–	–	–	35.66 (29.27)
Division	–	–	–	–	42.76 (29.61)
Total operations	–	–	–	47.85 (27.10)	46.18 (31.15)
Speeded writing	–	–	–	46.15 (26.23)	43.27 (25.87)
**Letter-knowledge^a^**					
Sounds	16.09 (10.48)	45.55 (4.18)	47.44 (2.94)	49.37 (2.99)	–
**SLRT-II reading fluency**					
Word		7.09 (4.94)	16.22 (9.64)	25.85 (12.23)	63.95 (24.13)
Pseudoword		11.32 (6.42)	17.44 (7.13)	21.33 (7.31)	39.98 (16.21)
Word (PR)	–	–	55.37 (27.40)	26.69 (22.30)	31.82 (28.79)
Pseudoword (PR)	–	–	50.22 (24.90)	26.70 (19.57)	34.07 (31.54)
Average (PR)	–	–	52.80 (24.59)	26.69 (20.14)	32.95 (29.61)

*^a^Sum of scores for items in lower and upper case (max score = 52); PR, percentile score. Raw scores are: number of correct items (Number-knowledge, Letter-knowledge), number of correct items within 1 min (SLRT-II).*

### Cognitive Assessments

**FIGURE 1:**
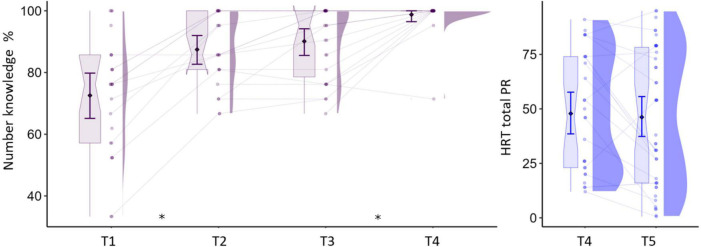
Numerical skills across measurements. *Y*-axis in left panel shows the percentage of correctly named numbers and in the right panel, the total percentile score for arithmetic operations. Asterisks indicate significant differences at *p* < 0.05 between test times. Boxplots show median and interquartile range. Error bars indicate mean and 95% CIs.

Cognitive tests were performed at each time point, depending on the test’s relevance to the specific grade level (Table 2). The scores for number knowledge and arithmetic skills are shown in Figure 1. Number knowledge was assessed from T1 to T4 and the test consisted of naming twenty-one numbers, including all single digits from 1 to 9, and numbers of up to three digits. Letter knowledge was assessed from T1 to T4 by asking participants to first identify the sound and then to name each letter from the Latin alphabet in German presented in blocks of upper and lower case letters (52 items in total). Basic arithmetic skills were assessed at T4 and T5 with several subtasks of the Heidelberger Rechentest (HRT; Haffner et al., 2005). The main subtasks were addition, subtraction, multiplication, comparison (fill gaps in simple formulas with the operators >, <, or =), and completion (fill numbers in simple formulas, e.g., 6 = _+3). A subtask with divisions was also included at T5. There were no normative scores available for multiplication and division at T4. Each subtask consisted of 40 items and raw scores were computed based on the number of correct responses within 2 min, except for the comparison task in which half the number of errors was subtracted from the correct responses. A total operations percentile score was calculated from normative values of the individual operation subtests, with higher values indicating better performance. In addition, the speeded writing subtask was used to assess visuomotor skills by requiring to copy numbers from a list of 60 items within 30 s.

Reading abilities were measured with the Salzburger Lese-und Rechtschreibtest (SLRT-II; Moll and Landerl, 2010). Fluency scores indicates the number of items correctly read within 1-min from lists of 156 items presented in eight columns. The lists contained either words or pseudowords, and for each there were two lists available: List A was used at T2 and T3, while list B was used at T4 and T5.

Finally, non-verbal IQ was estimated at T5 with the Reynolds Intellectual Assessment Scales (RIAS; Reynolds and Kamphaus, 2003), at T3 with the CFT1-R (Weiss and Osterland, 2013) and at T1 with the block design test of the Hamburg-Wechsler-Intelligenztest für Kinder (HAWIK-IV; Petermann and Petermann, 2010). The IQ estimate from the latest time point available was used (see note in Table 1). The description of rapid automated naming and phonological skills assessments are included in Supplementary Appendix A, “Cognitive Assessments”.

### Target Detection Task and Stimuli

**FIGURE 2:**
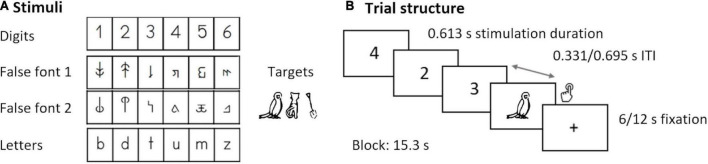
Target detection task. **(A)** Digits, false fonts, letters, and target stimuli. **(B)** Trial structure and presentation times.

The current analysis focuses on an implicit audiovisual target detection task performed during simultaneous EEG-fMRI recordings. The complete paradigm was divided in four parts (at T1–T4) and three parts (at T5) of 375 s, each of them presenting unimodal visual and auditory, as well as bimodal stimulation blocks separated by fixation periods of 6 or 12 s. Each time point included the presentation of the following three character types in separate parts: letters, digits, and false fonts. Participants were instructed to press a button whenever a target stimulus (picture or sound of an animal/tool) appeared on the screen. The task is illustrated in Figure 2. Our main analysis includes the unimodal visual conditions digits (DIG), letters (LET), and false fonts (FF), with a focus on the development of DIG vs. FF and DIG vs. LET differences. The analysis with a focus on LET vs. FF processing in a largely overlapping sample can be found in our previous publication (Fraga-González et al., 2021). Each condition consisted of four blocks with 15 items per condition (in total 54 trials and six target trials per condition). In the DIG condition, the stimuli were the Arabic numerals 1–6 (all participants were able to name these numbers with 100% accuracy). In the LET condition, the stimuli included the letters *b, d, m, t, u, z* from the Latin alphabet presented in “Swiss school” font (Figure 2). In the FF condition, the stimuli were two sets of characters matched in size and width with the LET characters, created by rearranging different parts of those letters (Karipidis et al., 2017). All stimuli were visually presented using goggles (VisuaStimDigital, Resonance Technology, Northridge, CA, United States) in black in the middle of a gray background (mean visual angles horizontally/vertically DIG: 3°/6.7; FF: 2.8/4.8°; LET: 2.8°/4.8°). The stimuli in each block were presented pseudorandomized, with a duration of 613 ms and followed by an interstimulus interval of either 331 or 695 ms. The task was programmed and presented using Presentation^®^ software (version 16.4)^1^ and the design was adjusted to find a compromise between the optimal designs for EEG and fMRI recordings, and to account for the attentional demands of young children.

### Electroencephalography Data Acquisition and Preprocessing

This section largely overlaps with that of our recent report as the same analysis procedure was followed (Fraga-González et al., 2021). EEG data were recorded at 1 kHz sampling rate using an MR-compatible 128-channel EEG system (Net Amps 400, EGI HydroCel Geodesic Sensor Net) during functional magnetic resonance imaging (fMRI) in a Philips Achieva 3T scanner (Philips Medical Systems, Best, Netherlands). Two additional electrodes placed on the chest registered the electrocardiogram (ECG). Impedances of the scalp electrodes were kept below 50 kΩ and the EEG system was synchronized with the scanner clock to reduce MR gradient artifacts. The recording reference was located at Cz and the ground electrode posterior to Cz. Vibrations were minimized by covering the electrodes with a bandage retainer net and turning off the helium pump and ventilation of the MRI scanner during image acquisition. Data were preprocessed with Vision Analyzer 2.1 (Brain Products GmbH, Munich, Germany). Electrodes with overall poor data quality or excessive artifacts were topographically interpolated (mean of 1.52 ± 0.54 and no more than 5 electrodes per subject). MR artifacts were removed using the average template subtraction method (Allen et al., 2000) and ballistocardiogram artifacts were corrected using sliding average template subtraction as implemented in Vision Analyzer 2.1. Additionally, continuous data were visually inspected to exclude periods with large artifacts like head movements. Subsequently, a band-pass filter of 0.1–30 Hz and 50 Hz Notch filter were applied and data were downsampled to 500 Hz. Then, we ran an independent component analysis (ICA) to remove components associated with blinks, eye movements, and residual ballistocardiogram artifacts. After artifact correction, data was visually inspected, electrodes located on the cheeks (E43, E48, E119, E120) were removed as they frequently contained major artifacts, and a 0.1 Hz high-pass filter was applied to minimize residual slow artifacts. Finally, data were re-referenced to the common average reference.

### Event-Related Potential Analysis

The continuous EEG data were epoched from −100 to 613 ms after stimulus onset. Epochs with amplitudes ±200 μV or segments visually identified as containing residual artifacts were discarded from analysis. Only participants with at least 20 epochs in each condition were included in the analysis. The mean (SD) number of epochs for the DIG condition were T1: 40.91 (8.93), T2: 44.23 (9.41), T3: 42.96 (9.09), T4: 37.96 (9.94), and T5: 47.93 (5.58). For the FF condition they were T1: 43.87 (5.22), T2: 43.45 (8.56), T3: 46.48 (4.31), T4: 41.56 (10.69), and T5: 47.69 (5.10). For the LET condition they were T1: 42.48 (10.09), T2: 42.55 (8.79), T3: 45.59 (7.55), T4: 41.00 (11.41), and T5: 47.31 (5.69). There were no significant differences between conditions in the number of segments in any of the time points, *ps* > 0.077. The N1 time window was defined using the global field power (GFP; Lehmann and Skrandies, 1980) of the ERP averaged across all three conditions and all subjects per time point. The interval was defined as ±30 ms around the GFP peak, i.e., around the second local maximum of the GFP, which corresponded with a typical N1 topography. The following intervals were defined independently at each time point based on the corresponding GFP peak to account for potential latency differences between measurements: T1: 198–258 ms, T2: 188–248 ms, T3: 190–250 ms, T4: 180–240 ms, and T5: 178–238 ms. Last, mean amplitudes of the N1 intervals were computed from the signal of the left occipito-temporal (LOT) cluster: E57, E58(=T5), E65, E70(=O1), E63, E64, E69, E68, E73 and the right occipito-temporal (ROT) cluster: E83(=O2), E90, E96(=T6), E100, E89, E95, E99, E88, E94. The clusters were defined based on visual inspection of topographies and previous studies (Pleisch et al., 2019).

### Statistical Analysis

The main analysis was performed by applying a linear mixed model (LMM) with a random intercept on N1 mean amplitudes and the fixed factors hemisphere (LOT, ROT), condition (DIG, LET, FF), and time point (T1, T2, T3, T4, T5). Since the goal of this study was to examine two types of digit sensitivity, we also planned and performed separate LMMs with digits and false fonts (coarse sensitivity), and with digits and letters (fine sensitivity) next to a separate LMM with letters and false fonts for completion. Potential interactions between time and condition were followed by *post hoc* tests for each time point. Note that digit sensitivity effects in these analyses would be reflected in negative *t* values, due the polarity of the N1 and testing for stronger (i.e., more negative) amplitudes for digits *minus* the control stimuli (letters or false fonts). The LMM was implemented with the function *lme* of the R package “Nlme” (Pinheiro et al., 2019). In our models, outliers were excluded if the normalized residuals exceeded the ±3 threshold, which resulted in exclusion of 7 data points (1% of the data) in the main model. Q-Q plots and predicted vs. residual plots were inspected to assess whether the data met the assumptions of normality and homoscedasticity.

Differences in topography between conditions during the N1 intervals were examined at each time point with a topographical analysis of variance (TANOVA; Strik et al., 1998) implemented with in-house scripts and Matlab functions (R2017a, MathWorks, Natick, MA, United States). The goal of this approach was to complement the analysis of N1 amplitudes by examining differences in the potential field distribution (topography) of activity. At each time point, voltage amplitudes across conditions were normalized to examine the activity distribution irrespective of strength. TANOVA uses non-parametric bootstrapping statistics per data point (5000 permutations, as recommended for an accurate estimate of the significance at the 1% level; Manly, 2007). The *p* values < 0.05 derived from these permutation tests indicate periods within the N1 interval where topographic differences between conditions were statistically significant.

Finally, we examined the relation of N1 digit sensitivity with the relevant cognitive skills as well as its potential usefulness as early predictor of literacy and numeracy. For this, we used non-parametric Spearman correlations to study the associations between neural, i.e., N1 digit-false font and N1 digit-letter amplitude differences over left and right hemispheres, and behavioral measures of interest, i.e., number knowledge at T1, and arithmetic and reading skills at T4 and T5 (R Core Team, 2017). The resulting rank correlation coefficients (Spearman’s ρ) assess the monotonic relationship between two variables. These correlations were examined before and after exclusion of outliers in the neural measures of interest (a maximum of five cases were excluded in T5). We used false discovery rate correction (FDR) to account for the number of tests (*n* = 36).

## Results

### Cognitive Performance

The analysis of cognitive tests yielded the expected significant time effects suggesting improvements in the raw scores for the main assessments (see Figure 1 and Table 2). Percentile scores indicating age-adjusted skill level are presented for the assessments and time points for which normative samples were available.

Number knowledge increased significantly from T1 to T4, as revealed by the corresponding linear mixed model analysis on the effect of measurement time, *F*(3,53) = 45.13, *p* < 0.001. The scores reached ceiling levels at T4, and pairwise comparisons showed significant differences between T1–T2 [*t*(53) = −6.38, *p* < 0.001] and T3–T4 [*t*(53) = −3.77, *p* = 0.002], but not in the period of T2–T3 (*p* = 0.652). A similar pattern was found for letter sound knowledge, with main effect of time [*F*(3,53) = 350.8, *p* < 0.001] and significant gains between T1–T2 [*t*(53) = −23.92, *p* < 0.001], but not between T2–T3 or T3–T4, *ps* > 0.102. The individual arithmetic skills increased from T4 to T5, as revealed by a significant effect of time on the HRT tests of addition [*F*(1,17) = 160.31, *p* < 0.001], subtraction [*F*(1,17) = 151.57, *p* < 0.001], comparison [*F*(1,17) = 135.91, *p* < 0.001], completion [*F*(1,17) = 65.57, *p* < 0.001], and the additional speeded writing subtest [*F*(1,17) = 142.33, *p* < 0.001]. There were no differences over time on the corresponding percentile scores, *ps* > 0.127. The number of participants performing <16th percentile in arithmetic operations (which may indicate poor math ability) at T1, T2, T3, T4, and T5 were 7, 5, 7, 8, and 12, respectively. Word and pseudoword reading were assessed from T2 to T5. Because different lists of items were used across time points (see section “Cognitive Assessments”), we tested T2 vs. T3 and T4 vs. T5 separately. The analysis showed a significant increase between T2 and T3 in the number of correctly read words [*F*(1,16) = 26.35 *p* < 0.001] and pseudowords [*F*(1,16) = 22.23, *p* < 0.001]. The same pattern was found between T4 and T5 for words [*F*(1,17) = 119.18, *p* < 0.001] and pseudowords [*F*(1,17) = 68.52, *p* < 0.001]. The percentile scores showed no statistically significant differences between all four time points, *ps* > 0.107. Finally, there were also significant gains in RAN and phonological skills. These analyses are reported in Supplementary Appendix B (section “Behavioral Assessments”) Supplementary Results, as they are not the focus of the current study.

### Event-Related Potential Analysis

Individual condition-wise visual N1 mean amplitude to digits, letters, and false fonts in the target detection task for each time point was used for statistical analyses. The current analysis focuses on the differences between digits and false fonts (coarse sensitivity) and the comparison of digits with letters (fine sensitivity; for the analysis focused on letter vs. false fonts see Fraga-González et al., 2021).

#### Development of N1 Responses to Digits

##### N1 Mean Amplitude

**FIGURE 3:**
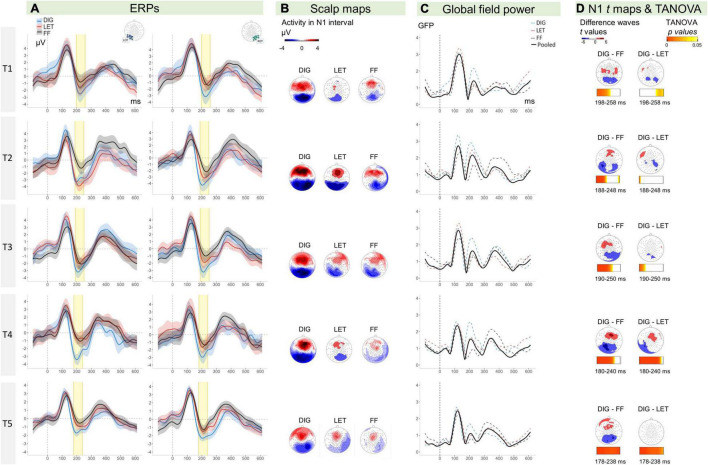
**(A)** ERPs (*y*-axis: μV, *x*-axis: time in ms) for digits (blue lines), letters (red lines), and false fonts (black lines) with ribbons indicating 95% CIs (within-subject). Left hemispheric amplitudes are depicted on the left side and right hemispheric amplitudes on the right. The N1 time-window is highlighted in yellow. **(B)** Scalp topographies for digits, letters, and false fonts. **(C)** GFP for the average of the three conditions (black line), digits (dashed blue line), letters (dashed red line), false fonts (dashed black line). **(D)** Scalp maps show the distribution of *t* values for the difference between conditions tested against zero (color steps display only values from 6 to 4 and 4 to 2, corresponding to ±*t* values significant for current sample sizes at *p* < 0.05). The horizontal bars below show *p* values resulting from TANOVA permutation tests of differences in topographies between conditions across each data point of the N1 time intervals (T1: 198–258 ms, T2: 188–248 ms, T3: 190–250 ms, T4: 180–240 ms, and T5: 178–238 ms). The bar color map from light to darker orange indicates *ps* ranging from 0.05 to 0; the blank periods indicate non-significant *ps* > 0.05. ERPs, event-related potentials; CIs, confidence intervals; GFP, global field power; TANOVA, topographic analysis of variance; DIG, digits; LET, letters; FF, false fonts; LOT, left occipito-temporal; ROT, right occipito-temporal.

**FIGURE 4:**
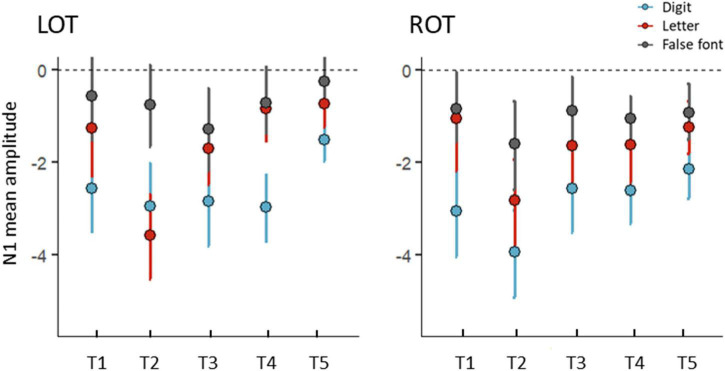
Mean amplitudes (μV) for the N1 interval over the five time points (T1, T2, T3, T4, and T5) for digits (blue), letters (red), and false fonts (black). Left (LOT) and right (ROT) electrode clusters are shown in separate panels. Error bars represent 95% CI. Normalized residuals from the main model exceeding ±3 threshold were excluded (7 data points).

The ERPs, visual N1 topographies and GFP waveforms per time point (T1 to T5), condition (DIG, LET, FF), and electrode cluster (LOT, ROT) are shown in Figures 3A–C. We performed a LMM analysis on N1 mean amplitudes with the factors time, condition and hemisphere (see sections “Event-Related Potential Analysis” and “Statistical Analysis” for N1 time range selection and model details). The analysis revealed a main effect of time [*F*(4,746) = 8.18, *p* < 0.001], indicating significant differences between the time points across conditions and hemispheres. Moreover, there was a main effect of condition [*F*(2,746) = 63.72, *p* < 0.001], indicating differences in amplitudes between the conditions over all time points and hemispheres. In addition, there was a significant interaction between the factors time and condition [*F*(8,746) = 2.40, *p* = 0.015], suggesting that these differences changed over time. No other effects were statistically significant, but there was a trend for a hemisphere effect, at *p* = 0.090. The mean amplitudes per condition, time and hemisphere are shown in Figure 4. A more detailed figure with box plots, individual data points and distribution is presented in the Supplementary Appendix Figure B1. This analysis was followed by LMMs for each pair of conditions.

Coarse and fine N1 digit sensitivity were examined with separate LMMs. The LMM using digits and false fonts (coarse sensitivity) showed an effect of time [*F*(4,480) = 3.22, *p* = 0.013], and a significant effect of condition [*F*(1,480) = 113.80, *p* < 0.001], but no significant interaction effects, *ps* > 0.111. The main effect of condition indicated stronger amplitudes for digits compared to false fonts over all time points (digits-false font *t* = −10.67 *p* < 0.001). The LMM with digits and letters (fine sensitivity) showed main effects of time [*F*(4,476) = 10.08, *p* < 0.001] and condition [*F*(1,476) = 41.36, *p* < 0.001], and a trend for an interaction between time and condition [*F*(8,476) = 2.13, *p* = 0.076]. This trend was explored by contrasts per time point, revealing significantly stronger amplitudes for digits at all time points (*ps* < 0.011) except at T2 (*p* = 0.545). The LMM with letters and false fonts (see also Fraga-González et al., 2021), showed significant main effects of time [*F*(4,477) = 6.22, *p* < 0.001] and condition [*F*(1,477) = 23.03, *p* < 0.001], together with a significant interaction between time and condition [*F*(8,477) = 3.73, *p* = 0.005]. The follow-up tests per time point comparing letters vs. false fonts only revealed stronger amplitudes for letters at T2 (*t* = −5.31, *p* < 0.001), all other *ps* > 0.151. The LMMs did not suggest an influence of electrode cluster, i.e., hemisphere on visual N1 sensitivity.

Additional topographical maps of *t*-tests on the difference between each pair of conditions are presented in Figure 3D. The figure shows a mainly bilateral distribution of the digit minus false font amplitudes. Exploratory *post hoc* comparisons per hemisphere and time point showed that this digit-false font difference was significant across time and hemispheres. The *t*-maps of the digit-letter differences did not show a consistent pattern of results across time, and suggest a potential left lateralization of N1 letter sensitivity at T4. Exploratory follow-up comparisons supported this by showing a significant digit-letter difference at T4 over the left hemisphere (*t* = −4.10, *p* < 0.001) but not over the right hemisphere (*p* = 0.150). This result should, however, be interpreted with caution since the main model within the electrode cluster of interest did not reveal significant interactions with the factor hemisphere.

**FIGURE 5:**
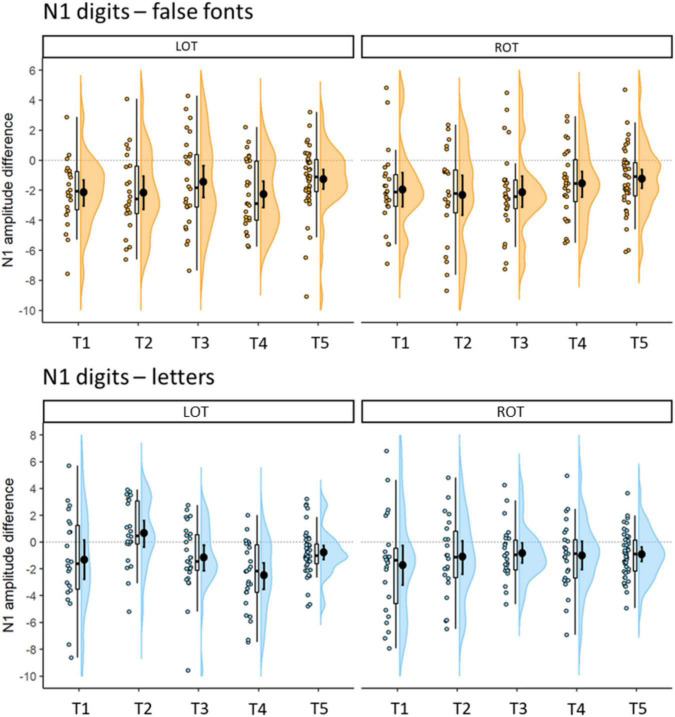
Differences in N1 mean amplitudes (μV) between digits and false fonts (coarse digit sensitivity: top plots) and digits and letters (fine digits sensitivity: bottom plots) per time point. Error bars within the density plot indicate mean and 95% CIs. More negative values indicate stronger negative amplitudes for digits than false fonts (top) or letters (bottom), respectively. Left (LOT) hemispheric cluster is depicted on the left panel, right (ROT) hemispheric cluster on the right.

Further details of the N1 differences between digits and false fonts and digits and letters, are presented in Figure 5. The percentage of participants showing stronger negativity for digits compared to false fonts in the left hemisphere were (T1) 82.61%, (T2) 77.27%, (T3) 62.96%, (T4) 77.78%, and (T5) 73.81%. In the right hemisphere the percentages were (T1) 86.96%, (T2) 77.27%, (T3) 85.19%, (T4) 74.07%, and (T5) 78.57%. Regarding the digits minus letter difference, the percentage of participants with stronger N1 amplitudes in the left hemisphere for digits were (T1) 60.87%, (T2) 31.82%, (T3) 66.67%, (T4) 84.62%, and (T5) 76.19%; in the right hemisphere the percentages were (T1) 78.26%, (T2) 63.64%, (T3) 74.07%, (T4) 69.23%, and (T5) 71.43%.

##### Topographic Analysis of Variance

An exploratory TANOVA compared the topographical distribution of activity between conditions at each time point within the N1 intervals. Amplitudes were normalized in this analysis so that the comparisons indicate differences in distribution independent from intensity of activity. The TANOVA results for the N1 time interval are plotted in Figure 3D. Significant differences in topography are indicated by *p* values of the permutation tests <0.05. We found significant differences between digit and false font map topographies at all time points in the target interval (N1). At T1, T2, and T3 these topographic differences were detected mainly at the beginning of the N1 interval while for T4 and T5, the topographic differences persisted almost over the whole N1 interval. For fine digit sensitivity (digit vs. letter comparisons), we did not find a clear period of topographic differences for the first three time points, but at T4 and T5 the topographies differed across most of the N1 intervals.

#### N1 Sensitivity to Digits and Cognitive Performance

The following analyses were performed separately with left and right hemisphere amplitudes. We first examined whether coarse (N1 digit-false font) or fine (N1 digit-letter) sensitivity to digits in kindergarten (T1) could predict later arithmetic or reading skills. Specifically, we focused on the latest score available (T5 when available, T4 otherwise) for the summary measure of arithmetic operations (HRT) and the mean of word and pseudoword reading scores (SLRT-II). The analyses revealed no significant associations between left or right N1 digit sensitivity at T1 and later arithmetic or reading skills (uncorrected *ps* > 0.072).

**FIGURE 6:**
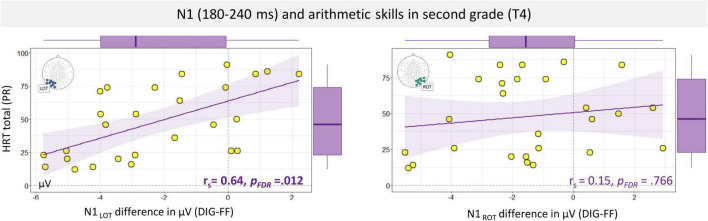
Spearman correlation and regression line of trends between total percentile score of arithmetic operations (HRT total) and N1 digits-false font amplitude differences in the left and right ventral-occipital electrodes. Box plots are displayed in the margins. LOT, left occipito-temporal; ROT, right occipito-temporal; DIG, digits; FF, false fonts; HRT, Heidelberger Rechentest; PR, percentile.

Next, the relationship between coarse and fine N1 digit sensitivity and arithmetic and reading skills was investigated within the different learning stages. The core cognitive measures were number knowledge at the first time point (T1) and arithmetic and reading skills in 2nd and 5th grade (T4 and T5). Here, it should be noted that performance in the number knowledge task was relatively high already in kindergarten and close to ceiling in subsequent measurements (T2 and T3), therefore the associations of N1 with number knowledge was only examined at T1; see Figure 1. The analysis of number knowledge yielded no statistically significant evidence for an association with coarse left or right N1 digit sensitivity at T1 (*p* = 0.139) but larger N1 fine (digit-letter) sensitivity over the left hemisphere was found to be associated with lower number knowledge. This result, however, did not survive multiple comparison corrections (Spearman ρ = 0.53, *p* = 0.009, *p_*FDR*_* = 0.108; *p*_*FDR*_ indicates a *p* value after False Discovery Rate correction for 36 tests). The analysis on arithmetic skills yielded significant associations between the age-adjusted level of arithmetic skills and the N1 amplitude differences at T4 (2nd grade; *N* = 27). Specifically, the coarse digit sensitivity in the N1 amplitudes over the left hemisphere showed a significant relation with the HRT percentile summary score for arithmetic operations (Spearman ρ = 0.64, *p* < 0.001, *p_*FDR*_* = 0.012). This association is presented in Figure 6. The direction of this relationship indicates larger coarse digit sensitivity over the left electrode cluster in children with lower arithmetic performance. *Post hoc* analyses of arithmetic subtests at T4, supported this association with coarse N1 digit sensitivity (addition and completion, *ps_*FDR*_* < 0.021, FDR corrected for 20 tests; see Supplementary Appendix Table B2). Interestingly, no significant associations were found between HRT arithmetic operations or reading skills and the N1 digit-false font difference over the right hemisphere, *ps* > 0.428. Regarding the fine sensitivity of N1 (digit-letter) at T4, there was no evidence for significant associations with reading or arithmetic skills, *ps* > 0.090. A table with all Spearman correlations is presented in Supplementary Appendix Table B1. The analysis at T5 yielded no significant associations with N1 coarse or fine sensitivity, although there was a trend for a negative association between right N1 coarse sensitivity and HRT total arithmetic operation scores (uncorrected *p* = 0.052) as well as reading scores (Spearman ρ = −0.44, *p* = 0.005, *p*_*FDR*_ = 0.090). Another trend for a negative association was found for left fine sensitivity and reading skills (uncorrected *p* = 0.054). The same pattern of results was found when using the raw scores. The analysis on raw scores is presented in Supplementary Appendix B section “N1 Amplitude Association With Arithmetic Skills (Raw Scores).”

## Discussion

The current study focused on N1 amplitude differences between digits, false fonts and letters across five time points in pre-school, middle and end of first grade, second grade, and fifth grade. Associations between coarse and fine N1 digit sensitivity (referring here to larger N1 amplitudes to digits vs. false fonts and digits vs. letters) and arithmetic and reading skills were also investigated. The results suggest a significant coarse sensitivity in the N1 responses to digits vs. false fonts that was present at all time points and an association of coarse N1 sensitivity with the arithmetic skills in 2nd grade. For the fine sensitivity of digits vs. letters a similar pattern of development emerged, with an absent fine sensitivity at T2, a developmental stage in which children are very much engaged in learning letters and letter-speech sound correspondences at school.

Our N1 amplitude analyses comparing digits to false fonts indicated that the strength of this coarse digit sensitivity seemed stable across time. This clearly contrasts the developmental trajectory of coarse letter sensitivity for which we previously characterized an inverted U-shaped trajectory (Fraga-González et al., 2021), only showing significant differences in the phase when children intensely practice their letter knowledge in first grade (T2 and T3), but not in pre-school or in second and fifth grade (T1, T4, and T5).

Our findings from the digit-letter comparisons were partially in accordance with those from Park et al. (2017), in which strings of letters, digits and false fonts were presented. In that study, the youngest group of 7-year-old children showed no difference between amplitudes over bilateral occipito-temporal regions for letters vs. digits, similar to our group at T2 in the current study, with a similar age (7.4 years). Here, it is important to note that the absence of the N1 fine digit sensitivity effect at T2, as compared to the fine digit sensitivity effects seen in all other time points, is explained by the particularly pronounced amplitudes to letters at T2, and not a change in the visual processing of digits. T2 corresponds to the time point during which children intensely train letter-speech sound correspondences (Fraga-González et al., 2021). In addition, Park et al. (2017) reported stronger amplitudes for letter strings relative to digits over the bilateral occipito-temporal scalp in 10-year-old children followed by left lateralized letter and right lateralized digit effects in 15-year-old children and adults. Even though the fine sensitivity effects were bilateral in our sample as well, the more pronounced amplitudes for digits over letters in children aged 7.7–11.4 years (T3–T5) contrast these findings. It seems likely that the presentation of strings, instead of single characters as in the current study, may account for this difference in processing and sensitivity. We expect consonant strings to be cognitively more demanding to process than single letters at these ages. Moreover, the difference between a single digit and a multi-digit number is conceptually not as large as between single letters and letter strings in terms of their associations. Unfortunately there were no measurements in younger children in the study of Park et al. (2017) for a further comparison of the effects prior to T2 in our study. Our results at T1 showing stronger amplitudes for digits compared to letters and false fonts are well in line with the results of Lochy and Schiltz (2019). In the latter study fast visual discrimination responses to digits compared to letters were found in young, 6-year-old children, and supports an early onset of specialized number processing. More importantly, our study also shows that coarse and fine visual N1 sensitivity to digits precedes the onset of specialized letter processing.

Although digits and letters may be comparable in terms of visual features and levels of exposure, they diverge in several attributes which may critically contribute to the development of brain systems for numeracy and literacy. Digits start being learned earlier than letters and convey a wide range of information, some of which may only be assimilated over many years of learning, e.g., complex mathematical properties. In the earlier years of numeracy, such as those covered by the current study, aspects like numerosity and parity become increasingly important (Miller and Gelman, 1983; Moeller et al., 2011). Importantly, these properties vary in the extent they may be automatically activated by different tasks (e.g., Cohen, 2009; Kallai and Tzelgov, 2012). Therefore, in the current paradigm, digits could be co-activating representations of different semantic properties over the time points, for example, when arithmetic skills emerge. According to the triple-code model of number processing, the non-verbal semantic representations of numbers (e.g., quantity) would mostly recruit parietal areas, while a verbal system for phonological and lexical representations would engage language areas (Dehaene et al., 2003). We would expect a network associated with the quantity system to be specific to digits, while areas of the verbal system and domain-general areas (e.g., visuo-spatial attention) required to perform a specific task may be shared by letters and digits. A meta-analysis reviewed the triple-code model and suggested the inclusion of additional regions that are likely to be detected in calculation or number tasks, such as the cingulate gyri, insula, and cerebellum (Arsalidou and Taylor, 2011). This meta-analysis also suggested a contribution of prefrontal areas involved in working memory processes and task difficulty.

If we consider the development of vOTC activations from an interactive perspective (Price and Devlin, 2011), it is possible that the interplay between specific number processing systems and domain general systems contribute to the current trajectory of digit processing, which diverges from the inverted U-shaped trajectory for letters. ERP studies using numerical Stroop tasks, showed parietal and occipito-temporal responses related to these additional cognitive demands and automatic processing of number semantic information (Szucs et al., 2007; Soltész et al., 2011b; Ben-Shalom et al., 2013). Previous studies showed that the N1 can be affected by general task demands and focus of attention (Hillyard and Anllo-Vento, 1998; Luck et al., 2000; Vogel and Luck, 2000; Yoncheva et al., 2010; Okumura et al., 2015). In future studies, manipulation over the different number properties elicited by the experimental task could help to clarify these contributions to the N1 ERP.

The trajectories of coarse and fine N1 sensitivity to digits in the current studied age range do not fit an inverted U-shaped trajectory that can be accommodated in the predictive coding framework for learning (Price and Devlin, 2011) or the cortical expansion-selection-renormalization model of skill learning (Wenger et al., 2017). An important point to discuss here, is the time period examined in the current study and the numeracy learning milestones captured by it. The results show that coarse and fine N1 sensitivity to digits are present already at kindergarten age (T1). As shown in Figure 1, knowledge of numbers is already high at this age, even though there is still a substantial improvement from T1 to T2. Consequently, the current data do not allow us to assess the emergence of N1 digit sensitivity with initial attainment of number knowledge, which presumably occurs earlier than the current T1 measurement. Even though the single-digits were already well known at T1, we only found an association that was not significant after multiple comparisons correction between fine (digits vs. letters) sensitivity and number knowledge at T1 over the left hemisphere, suggesting fine digit sensitivity especially in children who showed poor number knowledge. No association was found over the right hemisphere and it is likely that this association was mainly driven by differences in letter rather than digit processing. The lack of associations between N1 amplitudes over the right hemisphere and number knowledge may thus be due to the already advanced knowledge of digits at T1. Interestingly, our main results do not suggest a decay in neither coarse nor fine digit sensitivity across time points, contrasting previous findings for words and letters (Maurer et al., 2006; Fraga-González et al., 2021). Here, we should note that arithmetic operation skills are still intensively trained at T4 and T5. This period coincides with the onset of more fluent word and pseudoword decoding (see Table 2). It is possible that visual processing of digits remains functionally important to perform these arithmetic operations and that there is also automatic processing of new properties which are learned in parallel with arithmetic skills (e.g., numerosity, parity). Single letters on the other hand, may elicit weaker visual responses at this stage because they are mostly utilized as part of larger units like syllables and words, which carry much more complex phonological and semantic information. This could explain the sustained N1 sensitivity to digits in this period, in contrast with the observed decline of N1 letter sensitivity (Fraga-González et al., 2021).

In addition, we found significant associations between performance of simple arithmetic operations and coarse N1 digit sensitivity in second grade (T4), but not at a more advanced stage in fifth grade (T5) over the left hemisphere. Children with poorer arithmetic skills showed stronger N1 amplitudes for digits than false fonts over the left posterior electrodes. Due to the limited sample of 27 participants in this analysis, we can only provide a tentative interpretation of this result. The time point in 2nd grade in which children become firm with associating written characters with their spoken form, may indicate that children with poor arithmetic skills rely more on top-down input of their left lateralized language system to process digits (e.g., verbalize digits). It is known, that the left ventral occipito-temporal cortex is tightly coupled to various areas of the language network (Chen et al., 2019) and that these connections are emerging at a very early age (Saygin et al., 2016; Li et al., 2020; Yu et al., 2021). An alternative explanation could be that left vOTC responses to digits in children with poor arithmetic skills may be related to increased attention, necessary to process the digits. It has previously been shown that attention to digits modulates the activation in close by inferior temporal areas (Pollack and Price, 2019). Because we are examining digit processing in an implicit task, it is not possible to disentangle the contributions of different feedback processes in detail, this needs to be explored in future studies. Besides an uncorrected trend in the right hemisphere, there was no evidence for a reliable association of digit sensitivity at T5 with arithmetic skills, which may indicate that other systems for number processing, rather than the visual system, may be gaining functional importance as arithmetic skills and knowledge become more complex, i.e., the quantity or verbal system in the triple-code model (Dehaene, 1992).

Another point for discussion is the N1 lateralization. The ERP topographies and the lack of significant lateralization effects in our main model suggest that the N1 digit sensitivity was bilateral. The previous developmental report on the N1 to number strings suggested a late shift in lateralization toward more right lateralized N1 amplitudes for numbers that emerged in 15-year-olds and young adults (Park et al., 2017), which the current data did not capture. Our results are in contrast with the reported right lateralization of discrimination responses to digits embedded in letter streams using fast periodic visual stimulation in young children (Lochy and Schiltz, 2019). However, from previous reports on laterality of N1 to single characters it still remains unclear when this lateralization develops (Tarkiainen et al., 1999; Wong et al., 2005; Stevens et al., 2013; Fraga-González et al., 2021).

A limitation regarding generalizability of the current findings is that our sample consists of children with varying degree of reading skills and familial risk for dyslexia. There is evidence for comorbidity between dyslexia and dyscalculia and for shared features in the development of language and math skills (Butterworth et al., 2011; Peng et al., 2020). Although this sample may present the advantage of capturing a wider range of math achievement as it was found in our study, extrapolating these findings to the general population will require further studies with larger samples. Finally, as discussed above, the current visual target detection task cannot account for differences in attentional strategies and automatic processing of semantic features during performance.

### General Conclusion

We examined visual specialization for digits, reflected by stronger N1 ERP responses to digits compared to false fonts and letters. The results show that coarse and fine visual N1 sensitivity to digits is present already at pre-school and remains stable through primary school in children with varying familial risk for dyslexia and varying reading and math skills. This pattern of sensitivity is in marked contrast to the inverted U-shaped trajectory of sensitivity reported for single letter processing between the beginning of literacy acquisition and the consolidation of word reading towards the end of elementary school. The presence of coarse and fine sensitivity to digits in kindergarten clearly indicates that the specialization to number processing begins very early and before the onset of letter sensitivity in children. Even though it remains to be clarified when basic visual sensitivity to digits starts to emerge and how it interacts with developing verbal and quantity systems for numerical cognition during ontogeny, the stable digit sensitivity across middle childhood suggests that efficient number processing may rely on specialized visual number representations.

## Data Availability Statement

The data supporting the conclusions of this article will be made available by the authors, without undue reservation.

## Ethics Statement

The studies involving human participants were reviewed and approved by the Local Ethics Committee of the Canton of Zurich and neighboring Cantons in Switzerland. Written informed consent to participate in this study was provided by the participants’ legal guardian/next of kin.

## Author Contributions

GF-G conducted the statistical analysis, visualized the results, and wrote the first draft of the article. SB designed and coordinated the study. SB and IK co-wrote the article. GP, IK, and SB conceptualized the experiments. GP, IK, and SD collected the data. GF-G, GP, IK, and SD performed the data analysis. SW, DB, and SB provided infrastructure, critical feedback to research questions, experimental setup, and data analysis. All authors provided comments and contributed to the editing of the article.

## Conflict of Interest

SW has received in the last 5 years royalties from Thieme Hogrefe, Kohlhammer, Springer, Beltz. Her research work was supported in the last 5 years by the Swiss National Science Foundation (SNF), diff. EU FP7s, HSM Hochspezialisierte Medizin of the Kanton Zurich, Switzerland, Bfarm Germany, ZInEP, Hartmann Müller Stiftung, Olga Mayenfisch, Gertrud Thalmann, Vontobel, Unicentia, and Erika Schwarz Fonds. Outside professional activities and interests are declared under the link of the University of Zurich www.uzh.ch/prof/ssl-dir/interessenbindungen/client/web/. The remaining authors declare that the research was conducted in the absence of any commercial or financial relationships that could be construed as a potential conflict of interest.

## Publisher’s Note

All claims expressed in this article are solely those of the authors and do not necessarily represent those of their affiliated organizations, or those of the publisher, the editors and the reviewers. Any product that may be evaluated in this article, or claim that may be made by its manufacturer, is not guaranteed or endorsed by the publisher.

## Footnotes

1
www.neurobs.com

## References

[B1] Allen P. J. Josephs O. Turner R. (2000). A method for removing imaging artifact from continuous EEG recorded during functional MRI. *Neuroimage* 12 230–239. 10.1006/nimg.2000.0599 10913328

[B2] Amalric M. Dehaene S. (2016). Origins of the brain networks for advanced mathematics in expert mathematicians. *Proc. Natl. Acad. Sci. U.S.A.* 113 4909–4917. 10.1073/pnas.1603205113 27071124 PMC4983814

[B3] Araújo S. Faísca L. Bramão I. Reis A. Petersson K. M. (2015). Lexical and sublexical orthographic processing: an ERP study with skilled and dyslexic adult readers. *Brain Lang.* 141 16–27. 10.1016/j.bandl.2014.11.007 25528285

[B4] Arsalidou M. Taylor M. J. (2011). Is 2+2=4? Meta-analyses of brain areas needed for numbers and calculations. *Neuroimage* 54 2382–2393. 10.1016/j.neuroimage.2010.10.009 20946958

[B5] Bartelet D. Vaessen A. Blomert L. Ansari D. (2014). What basic number processing measures in kindergarten explain unique variability in first-grade arithmetic proficiency? *J. Exp. Child Psychol.* 117 12–28. 10.1016/j.jecp.2013.08.010 24128690

[B6] Ben-Shalom T. Berger A. Henik A. (2013). My brain knows numbers! - an ERP study of preschoolers’ numerical knowledge. *Front. Psychol.* 4:716. 10.3389/FPSYG.2013.00716 24155729 PMC3800772

[B7] Bentin S. Allison T. Puce A. Perez E. McCarthy G. (1996). Electrophysiological Studies of Face Perception in Humans. *J. Cogn. Neurosci.* 8 551–565. 10.1162/JOCN.1996.8.6.551 20740065 PMC2927138

[B8] Bentin S. Mouchetant-Rostaing Y. Giard M. H. Echallier J. F. Pernier J. (1999). ERP manifestations of processing printed words at different psycholinguistic levels: time course and scalp distribution. *J. Cogn. Neurosci.* 11 235–260. 10.1162/089892999563373 10402254

[B9] Brem S. Bach S. Kujala J. V. Maurer U. Lyytinen H. Richardson U. (2013). An electrophysiological study of print processing in kindergarten: the contribution of the visual N1 as a predictor of reading outcome. *Dev. Neuropsychol.* 38 567–594. 10.1080/87565641.2013.828729 24219696

[B10] Butterworth B. Varma S. Laurillard D. (2011). Dyscalculia: from brain to education. *Science* 332 1049–1053. 10.1126/science.1201536 21617068

[B11] Cantlon J. F. Pinel P. Dehaene S. Pelphrey K. A. (2011). Cortical representations of symbols, objects, and faces are pruned back during early childhood. *Cereb. Cortex* 21 191–199. 10.1093/CERCOR/BHQ078 20457691 PMC3000569

[B12] Chen L. Wassermann D. Abrams D. A. Kochalka J. Gallardo-Diez G. Menon V. (2019). The visual word form area (VWFA) is part of both language and attention circuitry. *Nat. Commun.* 10:5601. 10.1038/s41467-019-13634-z 31811149 PMC6898452

[B13] Cohen D. J. (2009). Integers do not automatically activate their quantity representation. *Psychon. Bull. Rev.* 16 332–336. 10.3758/PBR.16.2.332 19293103 PMC2658745

[B14] Dehaene S. (1992). Varieties of numerical abilities. *Cognition* 44 1–42. 10.1016/0010-0277(92)90049-N 1511583

[B15] Dehaene S. (2011). *The Number Sense: How the Mind Creates Mathematics, Revised and Updated Edition.* Oxford: Oxford University Press.

[B16] Dehaene S. Cohen L. (2007). Cultural recycling of cortical maps. *Neuron* 56 384–398. 10.1016/j.neuron.2007.10.004 17964253

[B17] Dehaene S. Cohen L. (2011). The unique role of the visual word form area in reading. *Trends Cogn. Sci.* 15 254–262. 10.1016/j.tics.2011.04.003 21592844

[B18] Dehaene S. Piazza M. Pinel P. Cohen L. (2003). Three parietal circuits for number processing. *Cogn. Neuropsychol.* 20 487–506. 10.1080/02643290244000239 20957581

[B19] Fraga-González G. Pleisch G. Di Pietro S. V. Neuenschwander J. Walitza S. Brandeis D. (2021). The rise and fall of rapid occipito-temporal sensitivity to letters: transient specialization through elementary school. *Dev. Cogn. Neurosci.* 49:100958. 10.1016/j.dcn.2021.100958 34010761 PMC8141525

[B20] Fraga-González G. Žarić G. Tijms J. Bonte M. Blomert L. Van der Molen M. W. (2014). Brain-potential analysis of visual word recognition in dyslexics and typically reading children. *Front. Hum. Neurosci.* 8:474. 10.3389/fnhum.2014.00474 25071507 PMC4075352

[B21] Friston K. J. (2010). The free-energy principle: a unified brain theory? *Nat. Rev. Neurosci.* 11 127–138. 10.1038/nrn2787 20068583

[B22] Grotheer M. Herrmann K. H. Kovács G. (2016). Neuroimaging evidence of a bilateral representation for visually presented numbers. *J. Neurosci.* 36 88–97. 10.1523/JNEUROSCI.2129-15.2016 26740652 PMC6601794

[B23] Guillaume M. Poncin A. Schiltz C. Van Rinsveld A. (2020). Measuring spontaneous and automatic processing of magnitude and parity information of Arabic digits by frequency-tagging EEG. *Sci. Rep.* 10:22254. 10.1038/s41598-020-79404-w 33335293 PMC7747728

[B24] Haffner J. Baro K. Parzer P. Resch F. (2005). *Heidelberger Rechentest (HRT 1-4). Erfassung Mathematischer Basiskompetenzen im Grundschulalter.* Hogrefe: Göttingen.

[B25] Hillyard S. A. Anllo-Vento L. (1998). Event-related brain potentials in the study of visual selective attention. *Proc. Natl. Acad. Sci. U.S.A.* 95 781–787. 10.1073/PNAS.95.3.781 9448241 PMC33798

[B26] Kallai A. Y. Tzelgov J. (2012). The place-value of a digit in multi-digit numbers is processed automatically. *J. Exp. Psychol. Learn. Mem. Cogn.* 38 1221–1233. 10.1037/a0027635 22449132

[B27] Karipidis I. I. Pleisch G. Brandeis D. Roth A. Röthlisberger M. Schneebeli M. (2018). Simulating reading acquisition: the link between reading outcome and multimodal brain signatures of letter–speech sound learning in prereaders. *Sci. Rep.* 8:7121. 10.1038/s41598-018-24909-8 29740067 PMC5940897

[B28] Karipidis I. I. Pleisch G. Röthlisberger M. Hofstetter C. Dornbierer D. Stämpfli P. (2017). Neural initialization of audiovisual integration in prereaders at varying risk for developmental dyslexia. *Hum. Brain Mapp.* 38 1038–1055. 10.1002/hbm.23437 27739608 PMC6866885

[B29] Kucian K. Von Aster M. Loenneker T. Dietrich T. Martin E. (2008). Development of neural networks for exact and approximate calculation: a fMRI study. *Dev. Neuropsychol.* 33 447–473. 10.1080/87565640802101474 18568899

[B30] Lau N. T. T. Merkley R. Tremblay P. Zhang S. De Jesus S. Ansari D. (2021). Kindergarteners’ symbolic number abilities predict nonsymbolic number abilities and math achievement in grade 1. *Dev. Psychol.* 57 471–488. 10.1037/DEV0001158 33630621

[B31] Lefly D. L. Pennington B. F. (2000). Reliability and validity of the adult reading history questionnaire. *J. Learn. Disabil.* 33 286–296. 10.1177/002221940003300306 15505966

[B32] Lehmann D. Skrandies W. (1980). Reference-free identification of components of checkerboard-evoked multichannel potential fields. *Electroencephalogr. Clin. Neurophysiol.* 48 609–621. 10.1016/0013-4694(80)90419-8 6155251

[B33] Li J. Osher D. E. Hansen H. A. Saygin Z. M. (2020). Innate connectivity patterns drive the development of the visual word form area. *Sci. Rep.* 10:18039. 10.1038/s41598-020-75015-7 33093478 PMC7582172

[B34] Lochy A. Schiltz C. (2019). Lateralized neural responses to letters and digits in first graders. *Child Dev.* 90 1866–1874. 10.1111/cdev.13337 31657009 PMC6916599

[B35] Luck S. J. Woodman G. F. Vogel E. K. (2000). Event-related potential studies of attention. *Trends Cogn. Sci.* 4 432–440. 11058821 10.1016/s1364-6613(00)01545-x

[B36] Manly B. F. J. (2007). *Randomization, Bootstrap and Monte Carlo Methods in Biology.* Boca Raton, FL: Chapman & Hall/CRC.

[B37] Maurer U. Brem S. Kranz F. Bucher K. Benz R. Halder P. (2006). Coarse neural tuning for print peaks when children learn to read. *Neuroimage* 33 749–758. 10.1016/j.neuroimage.2006.06.025 16920367

[B38] McCaskey U. Von Aster M. Maurer U. Martin E. O’Gorman Tuura R. Kucian K. (2018). Longitudinal brain development of numerical skills in typically developing children and children with developmental dyscalculia. *Front. Hum. Neurosci.* 11:629. 10.3389/fnhum.2017.00629 29354041 PMC5758587

[B39] Mehringer H. Fraga-González G. Pleisch G. Röthlisberger M. Aepli F. Keller V. (2020). (Swiss) GraphoLearn: an app-based tool to support beginning readers. *Res. Pract. Technol. Enhanc. Learn.* 15:5. 10.1186/s41039-020-0125-0 32175013 PMC7048874

[B40] Miller K. Gelman R. (1983). The child’s representation of number: a multidimensional scaling analysis. *Child Dev.* 54:1470. 10.2307/1129809

[B41] Moeller K. Pixner S. Zuber J. Kaufmann L. Nuerk H. C. (2011). Early place-value understanding as a precursor for later arithmetic performance-A longitudinal study on numerical development. *Res. Dev. Disabil.* 32 1837–1851. 10.1016/j.ridd.2011.03.012 21498043

[B42] Moll K. Landerl K. (2010). *SLRT-II: Lese-und Rechtschreibtest.* Berne: Huber.

[B43] Nobre A. C. Allison T. McCarthy G. (1994). Word recognition in the human inferior temporal lobe. *Nature* 372 260–263. 7969469 10.1038/372260a0

[B44] Okumura Y. Kasai T. Murohashi H. (2015). Attention that covers letters is necessary for the left-lateralization of an early print-tuned ERP in Japanese hiragana. *Neuropsychologia* 69 22–30. 10.1016/j.neuropsychologia.2015.01.026 25613647

[B45] Park J. Chiang C. Brannon E. M. Woldorff M. G. (2014). Experience-dependent hemispheric specialization of letters and numbers is revealed in early visual processing. *J. Cogn. Neurosci.* 26 2239–2249. 10.1162/jocn_a_00621 24669789 PMC4261939

[B46] Park J. Hebrank A. Polk T. A. Park D. C. (2012). Neural dissociation of number from letter recognition and its relationship to parietal numerical processing. *J. Cogn. Neurosci.* 24 39–50. 10.1162/jocn_a_00085 21736455 PMC3357212

[B47] Park J. van den Berg B. Chiang C. Woldorff M. G. Brannon E. M. (2017). Developmental trajectory of neural specialization for letter and number visual processing. *Dev. Sci.* 21:e12578. 10.1111/desc.12578 28681391

[B48] Peng P. Lin X. Ünal Z. E. Lee K. Namkung J. Chow J. (2020). Examining the mutual relations between language and mathematics: a meta-analysis. *Psychol. Bull.* 146 595–634. 10.1037/bul0000231 32297751

[B49] Petermann F. Petermann U. (2010). *HAWIK-IV: Hamburg-Wechsler-Intelligenztest für Kinder-IV; Manual; Übersetzung und Adaption der WISC-IV von David Wechsler.* Bern: Huber.

[B50] Peters L. de Smedt B. Op de Beeck H. P. (2015). The neural representation of arabic digits in visual cortex. *Front. Hum. Neurosci.* 9:517. 10.3389/fnhum.2015.00517 26441613 PMC4585091

[B51] Pinheiro J. Bates D. DebRoy S. Sarkar D. R Core Team (2019). *nlme: Linear and Nonlinear Mixed Effects Models.* Available online at: https://cran.r-project.org/package=nlme (accessed June 8, 2021).

[B52] Pleisch G. Karipidis I. I. Brauchli C. Röthlisberger M. Hofstetter C. Stämpfli P. (2019). Emerging neural specialization of the ventral occipitotemporal cortex to characters through phonological association learning in preschool children. *Neuroimage* 189 813–831. 10.1016/j.neuroimage.2019.01.046 30677503

[B53] Pollack C. Price G. R. (2019). Neurocognitive mechanisms of digit processing and their relationship with mathematics competence. *Neuroimage* 185 245–254. 10.1016/J.NEUROIMAGE.2018.10.047 30342974 PMC7845410

[B54] Price C. J. Devlin J. T. (2011). The interactive account of ventral occipitotemporal contributions to reading. *Trends Cogn. Sci.* 15 246–253. 10.1016/j.tics.2011.04.001 21549634 PMC3223525

[B55] R Core Team (2017). *R: A Language and Environment for Statistical Computing.* Vienna: R Foundation for Statistical Computing.

[B56] Reynolds C. R. Kamphaus R. W. (2003). *Reynolds Intellectual Assessment Scales: Professional Manual.* Lutz, FL: PAR.

[B57] Saygin Z. M. Osher D. E. Norton E. S. Youssoufian D. A. Beach S. D. Feather J. (2016). Connectivity precedes function in the development of the visual word form area. *Nat. Neurosci.* 19 1250–1255. 10.1038/nn.4354 27500407 PMC5003691

[B58] Shum J. Hermes D. Foster B. L. Dastjerdi M. Rangarajan V. Winawer J. (2013). A brain area for visual numerals. *J. Neurosci.* 33 6709–6715. 10.1523/JNEUROSCI.4558-12.2013 23595729 PMC3970733

[B59] Skagenholt M. Skagerlund K. Träff U. (2021). Neurodevelopmental differences in child and adult number processing: an fMRI-based validation of the triple code model. *Dev. Cogn. Neurosci.* 48:100933. 10.1016/J.DCN.2021.100933 33582487 PMC7890357

[B60] Sokolowski H. M. Fias W. Mousa A. Ansari D. (2017). Common and distinct brain regions in both parietal and frontal cortex support symbolic and nonsymbolic number processing in humans: a functional neuroimaging meta-analysis. *Neuroimage* 146 376–394. 10.1016/j.neuroimage.2016.10.028 27769786

[B61] Soltész F. Goswami U. White S. Szucs D. (2011a). Executive function effects and numerical development in children: behavioural and ERP evidence from a numerical Stroop paradigm. *Learn. Individ. Differ.* 21 662–671. 10.1016/j.lindif.2010.10.004

[B62] Soltész F. White S. Szucs D. (2011b). Event-related brain potentials dissociate the developmental time-course of automatic numerical magnitude analysis and cognitive control functions during the first three years of primary school. *Dev. Neuropsychol.* 36 682–701. 10.1080/87565641.2010.549982 21761993

[B63] Starrfelt R. Behrmann M. (2011). Number reading in pure alexia-A review. *Neuropsychologia* 49 2283–2298. 10.1016/j.neuropsychologia.2011.04.028 21554892

[B64] Stevens C. McIlraith A. Rusk N. Niermeyer M. Waller H. (2013). Relative laterality of the N170 to single letter stimuli is predicted by a concurrent neural index of implicit processing of letter names. *Neuropsychologia* 51 667–674. 10.1016/j.neuropsychologia.2012.12.009 23274433

[B65] Strik W. K. Fallgatter A. J. Brandeis D. Pascual-Marqui R. D. (1998). Three-dimensional tomography of event-related potentials during response inhibition: evidence for phasic frontal lobe activation. *Electroencephalogr. Clin. Neurophysiol.* 108 406–413. 10.1016/S0168-5597(98)00021-5 9714383

[B66] Szucs D. Soltész F. (2008). The interaction of task-relevant and task-irrelevant stimulus features in the number/size congruency paradigm: an ERP study. *Brain Res.* 1190 143–158. 10.1016/j.brainres.2007.11.010 18076868

[B67] Szucs D. Soltész F. Jármi É Csépe V. (2007). The speed of magnitude processing and executive functions in controlled and automatic number comparison in children: an electro-encephalography study. *Behav. Brain Funct.* 3:23. 10.1186/1744-9081-3-23/FIGURES/8 17470279 PMC1872027

[B68] Tanaka J. W. Curran T. (2001). A neural basis for expert object recognition. *Psychol. Sci.* 12 43–47. 11294227 10.1111/1467-9280.00308

[B69] Tarkiainen A. Helenius P. Hansen P. C. Cornelissen P. L. Salmelin R. (1999). Dynamics of letter string perception in the human occipitotemporal cortex. *Brain* 122(Pt. 1), 2119–2132. 10545397 10.1093/brain/122.11.2119

[B70] Thesen T. McDonald C. R. Carlson C. Doyle W. Cash S. Sherfey J. (2012). Sequential then interactive processing of letters and words in the left fusiform gyrus. *Nat. Commun.* 3:1284. 10.1038/ncomms2220 23250414 PMC4407686

[B71] Thierry G. Pegna A. J. Dodds C. Roberts M. Basan S. Downing P. (2006). An event-related potential component sensitive to images of the human body. *Neuroimage* 32 871–879. 10.1016/J.NEUROIMAGE.2006.03.060 16750639

[B72] Vogel E. K. Luck S. J. (2000). The visual N1 component as an index of a discrimination process. *Psychophysiology* 37 190–203. 10731769

[B73] Wang F. Karipidis I. I. Pleisch G. Fraga-González G. Brem S. (2020). Development of print-speech integration in the brain of beginning readers with varying reading skills. *Front. Hum. Neurosci.* 14:289. 10.3389/fnhum.2020.00289 32922271 PMC7457077

[B74] Weiss R. H. Osterland J. (2013). *CFT 1-R Grundintelligenztest Skala 1 - Revision.* Göttingen: Hogrefe.

[B75] Wenger E. Brozzoli C. Lindenberger U. Lövdén M. (2017). Expansion and renormalization of human brain structure during skill acquisition. *Trends Cogn. Sci.* 21 930–939. 10.1016/j.tics.2017.09.008 29149999 PMC5697733

[B76] Wong A. C. N. Gauthier I. Woroch B. DeBuse C. Curran T. (2005). An early electrophysiological response associated with expertise in letter perception. *Cogn. Affect. Behav. Neurosci.* 5 306–318. 10.3758/cabn.5.3.306 16396092

[B77] Yeo D. J. Wilkey E. D. Price G. R. (2017). The search for the number form area: a functional neuroimaging meta-analysis. *Neurosci. Biobehav. Rev.* 78 145–160. 10.1016/j.neubiorev.2017.04.027 28467892

[B78] Yoncheva Y. N. Blau V. C. Maurer U. McCandliss B. D. (2010). Attentional focus during learning impacts N170 ERP responses to an artificial script. *Dev. Neuropsychol.* 35 423–445. 10.1080/87565641.2010.480918 20614358 PMC4365954

[B79] Yu X. Ferradal S. L. Sliva D. D. Dunstan J. Carruthers C. Sanfilippo J. (2021). Functional connectivity in infancy and toddlerhood predicts long-term language and preliteracy outcomes. *Cereb. Cortex* Online ahead of print, 10.1093/CERCOR/BHAB230 34347052 PMC10847903

