# Supplementary material for: Visual Occipito-Temporal N1 Sensitivity to Digits Across Elementary School
Source: Front Hum Neurosci. 2022 Jul 26;16:887413. doi: 10.3389/fnhum.2022.887413 (PMC9360418; doi:10.3389/fnhum.2022.887413)
Supplement: Supplementary file 1 [file Data_Sheet_1.docx]

**Supplementary Informatiom**

# Appendix A: supplementary methods

## Overview of available data

Fig. A.1. The tile plots on the left show longitudinal data availability for the contrasts digits vs false fonts, and for all three conditions. Columns represent participants and rows measurement time points (TP). Colored tiles indicate cases available for analysis and the x-axis labels indicate sample sizes. The bar plots summarize the sample size (N) per TP and the N depending of the number of measurements available (middle and right column of plots, respectively).


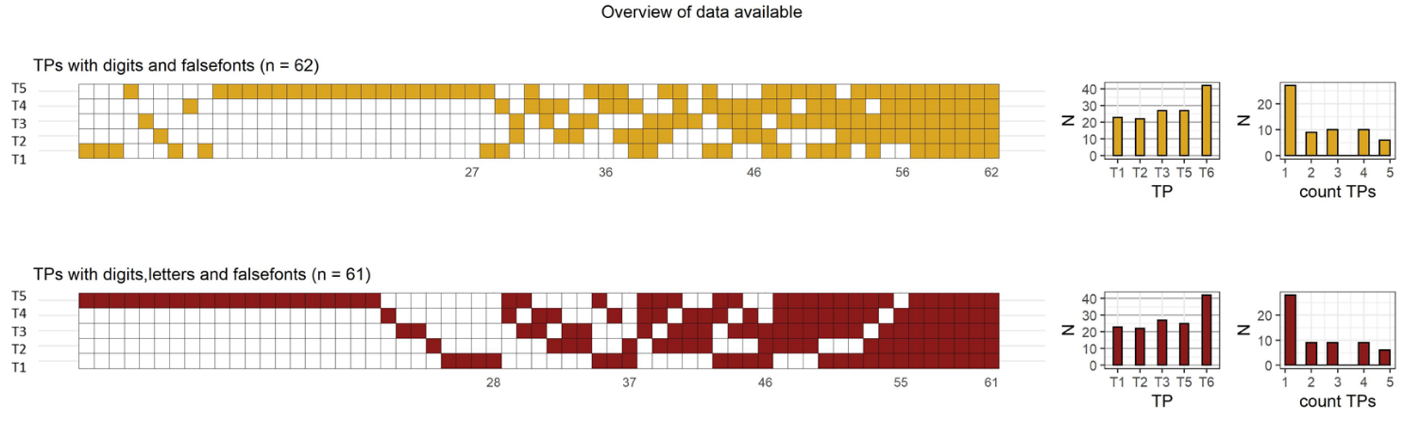


## Cognitive assessments

Phonological processing was assessed with subtests of two standardized tests (some subtests were not assessed after T3 as they were designed for children up to first grade). We used the rhyme and initial sound categorization subtests from TEPHOBE (Mayer, 2011). In the rhyme subtest, children were asked to select two rhyming words out of four orally presented words, whereas in the initial sound categorization subtest they were asked to select two out of four words starting with the same speech sound. Both subtests consist of seven trials and pictures of the words were provided to minimize working memory load. In addition, we used several subtests from the Basiskompetenzen für Lese-Rechtschreibleistungen (BAKO; Stock, Marx, & Schneider, 2013). In the phoneme deletion subtest, the children had to detect and delete the initial speech sound of a word or pseudoword and pronounce it aloud without that sound (max. seven trials). In pseudoword segmentation, children had to segment auditorily presented pseudowords by vocalizing each phoneme separately while moving tokens representing each phoneme towards the experimenter (max. eight trials). In the vowel substitution subtest, children had to repeat words while replacing all instances of the vowel [a] with [ɪ] (12 trials). The number of correct trials were used as raw scores in all subtests from BAKO test.

In addition, we assessed the rapid automatized naming (RAN) subtests of letters, numbers, colors, and objects of the “Test zur Erfassung der phonologischen Bewusstheit und der Benennungsgeschwindigkeit” (TEPHOBE; Mayer, 2011).

# Appendix B: Supplementary results

## Behavioral assessments

RAN skills showed improvements over time. RAN objects and colors were tested from T1 to T5, and RAN letters and digits from T2 to T5. The linear mixed model analysis showed a significant effect of time for RAN colors (*F* (4,74) = 49.68, *p <* 0.001), RAN letters (*F* (3,58) = 101.86, *p <* 0.001), RAN numbers (*F* (3,58) = 93.42, *p <* 0.001), and RAN objects (*F* (4,75) = 105.03, *p <* 0.001). The pairwise comparisons for RAN colors showed significant differences for T1-T2 (*t* (74) = -3.45, *p* = 0.008) and T4-T5 (*t* (74) = -6.19, *p* < 0.001), and a trend for T3-T4 (*t* (74) = -2.72, *p* = 0.060), while the difference for T2-T3 was not statistically significant (*p* = 0.710). For RAN letters, the analysis yielded significant differences for T2-T3 (*t* (58) = -3.92, *p* = 0.001) and T4-T5 (*t* (58) = -11.89, *p <* 0.001) but not for T3-T4, *p* = 0.439. For RAN numbers we found statistically significant differences for T3-T4 (*t* (58) = -3.33, *p* = 0.008) and T4-T5 (*t* (58) = -10.63, *p <* 0.001), but not for T2-T3, *p* = 0.175. Lastly, the comparisons for RAN objects yielded statistically significant differences for T3-T4 (*t* (75) = -4.61, *p* < 0.001) and T4-T5 (*t* (75) = -9.82, *p* < 0.001), all other *ps* > 0.129.

Regarding phonological processing assessments, we found statistically significant improvements in TEPHOBE initial sound categorization over time, *F* (2,30) = 51.48, *p <* 0.001. The gains were significant from T1 to T2 (*t* (30) = -7.60, *p <* 0.001), the T2-T3 comparison did not yield a significant effect, *p* = 0.157. Change with time in TEPHOBE rhyme scores was not statistically significant (*p =* 0.406). We should note that performance in TEPHOBE tests (with just 7 items) was close to ceiling levels already at T2. This was not the case for BAKO tests. The BAKO phoneme deletion test showed gains with time (*F* (2,30) = 36.28, *p* < 0.001), that were significant for the T1-T2 comparison (*t* (30) = -6.68, *p* < 0.001), but not T2-T3, *p* = 0.479. For BAKO pseudoword segmentation, the main effect of time (*F* (2,30) = 46.90, *p* < 0.001) was followed by significant effect for T1-T2 (*t* (30) = -6.52, *p* < 0.001) and T2-T3 (*t* (30) = -2.79, *p* = 0.024). The BAKO vowel replacement was tested at four timepoints (T1 to T4); statistical significance was found in the overall changes over time (*F (1,* 53) = 75.81, *p* < 0.001) and the T1-T2 comparison (*t* (53) = -10.53, *p* < 0.001), but not for T2-T3 and T3-T4 comparisons (*ps* > 0.213).

Finally, word and pseudoword reading were assessed from T2 to T5. Because different lists of items were used across time points (see section Cognitive Assessments), we tested T2-T3 and T4-T5 separately and percentile scores were only compared between the latest two measurements. The analysis in the first period showed a significant increase in the number of correctly read words (*F* (1,16) = 26.35 *p <* 0.001) and pseudowords (*F* (1,16) = 22.23, *p* < 0.001) between T2 and T3. The comparisons of raw scores in the T4-T5 period showed significant gains for words (*F* (1,17) = 119.18, *p* < 0.001) and pseudowords (*F* (1,17) = 68.52, *p* < 0.001). The analysis on percentile scores showed no statistically significant gains in words, pseudowords or the average percentile, *ps* > 0.107. Overall, the average percentile scores were relatively low in our sample of children at risk for dyslexia, but there was large variability between individuals. From all the children, the mean (SD; range) percentile reading scores for T4 and T5 were 26.69 (20.14; 1.25-72.75) and 32.95 (29.61; 1-96), respectively.

## N1 mean amplitudes for letters, digits and false fonts

Fig.B.1. N1 mean amplitudes (µV) for digits (DIG: blue), letters (LET: red) and false fonts (FF: black) for left and right clusters (top and bottom row, respectively) per measurement time. Scatter points show individual means. Error bars inside boxplots show the mean and 95 % CI. LOT=left occipitotemporal; ROT = right occipito-temporal.


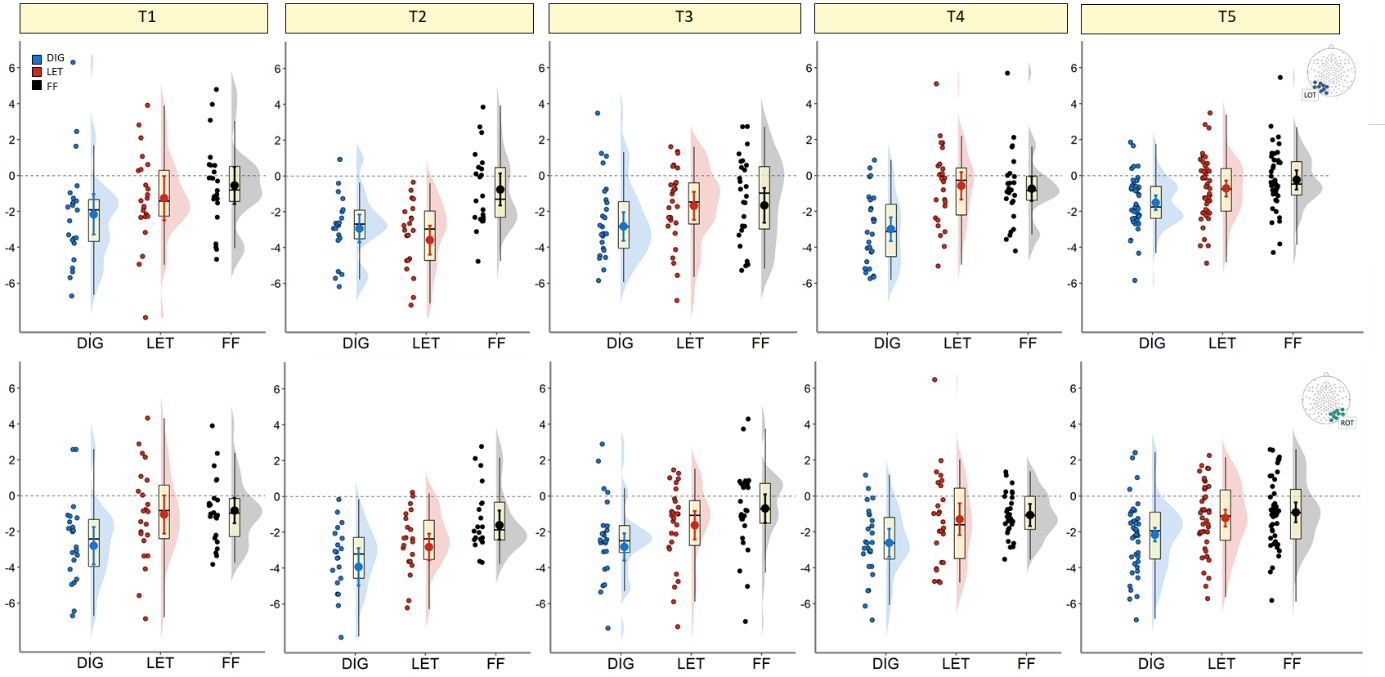


## N1 amplitude association with arithmetic skills (Spearman correlations)

| **Table B.1.** Spearman correlations between N1 sensitivity and cognitive measures. | | | | | | | | |
| --- | --- | --- | --- | --- | --- | --- | --- | --- |
| Time point | N1 sensitivity | Hemisphere | Cognitive measure | *n* | Spearman ρ | *p* | *p_FDR_* |  |
|  |  |  |  |  |  |  |  |  |
| T1 | digit-false font | left | Number knowledge | 21 | -0.33 | 0.139 | 0.385 |  |
|  |  |  | HRT total (T4) | 20 | -0.10 | 0.682 | 0.877 |  |
|  |  |  | SLRT-II mean (T4) | 20 | -0.36 | 0.119 | 0.357 |  |
|  |  |  | HRT total (T5) | 15 | -0.14 | 0.629 | 0.839 |  |
|  |  |  | SLRT-II mean (T5) | 13 | -0.16 | 0.59 | 0.820 |  |
|  |  | right | Number knowledge | 20 | -0.08 | 0.744 | 0.893 |  |
|  |  |  | HRT total (T4) | 20 | 0.19 | 0.432 | 0.766 |  |
|  |  |  | SLRT-II mean (T4) | 20 | -0.23 | 0.33 | 0.743 |  |
|  |  |  | HRT total (T5) | 15 | -0.47 | 0.076 | 0.353 |  |
|  |  |  | SLRT-II mean (T5) | 13 | -0.19 | 0.529 | 0.820 |  |
|  |  |  |  |  |  |  |  |  |
|  | digit-letter | left | Number knowledge | *23* | *0.53* | *0.009* | *0.108* |  |
|  |  |  | HRT total (T4) | 22 | 0.35 | 0.108 | 0.353 |  |
|  |  |  | SLRT-II mean (T4) | 22 | 0.39 | 0.072 | 0.353 |  |
|  |  |  | HRT total (T5) | 17 | 0.17 | 0.503 | 0.820 |  |
|  |  |  | SLRT-II mean (T5) | 15 | 0.03 | 0.909 | 0.963 |  |
|  |  | right | Number knowledge | 22 | 0.06 | 0.787 | 0.914 |  |
|  |  |  | HRT total (T4) | 21 | 0.24 | 0.291 | 0.698 |  |
|  |  |  | SLRT-II mean (T4) | 21 | -0.12 | 0.592 | 0.820 |  |
|  |  |  | HRT total (T5) | 16 | -0.16 | 0.559 | 0.820 |  |
|  |  |  | SLRT-II mean (T5) | 14 | -0.11 | 0.714 | 0.886 |  |
|  |  |  |  |  |  |  |  |  |
| T4 | digit-false font | left | SLRT-II mean | 27 | -0.02 | 0.936 | 0.963 |  |
|  |  |  | HRT total | **27** | **0.64** | **<0.001** | **0.012** |  |
|  |  | right | SLRT-II mean | 27 | -0.16 | 0.428 | 0.766 |  |
|  |  |  | HRT total | 27 | 0.15 | 0.447 | 0.766 |  |
|  | digit-letters | left | SLRT-II mean | 26 | <0.01 | 0.985 | 0.985 |  |
|  |  |  | HRT total | 26 | 0.34 | 0.091 | 0.353 |  |
|  |  | right | SLRT-II mean | 24 | 0.17 | 0.415 | 0.766 |  |
|  |  |  | HRT total | 24 | 0.27 | 0.194 | 0.499 |  |
|  |  |  |  |  |  |  |  |  |
| T5 | digit-false font | left | HRT total | 40 | -0.04 | 0.826 | 0.929 |  |
|  |  |  | SLRT-II mean | 40 | -0.31 | 0.054 | 0.353 |  |
|  |  | right | HRT total | 39 | -0.31 | 0.052 | 0.353 |  |
|  |  |  | SLRT-II mean | *39* | *-0.44* | *0.005* | *0.090* |  |
|  | digit-letter | left | HRT total | 36 | 0.15 | 0.373 | 0.766 |  |
|  |  |  | SLRT-II mean | 36 | -0.02 | 0.913 | 0.963 |  |
|  |  | right | HRT total | 42 | -0.26 | 0.096 | 0.353 |  |
|  |  |  | SLRT-II mean | 42 | -0.26 | 0.099 | 0.353 |  |
|  |  |  |  |  |  |  |  |  |
| FDR = False Discovery Rate correction for 36 tests; Percentile scores except for number knowledge. HRT = Heidelberger Rechentest (arithmetic skills); SLRT-II = Salzburger Lese- und Rechtschreibtest II (reading skills). Bold text indicates results at *p_FDR_* < 0.05; Italic text indicates results at *p* <0.05. | | | | | | | | |

| **Table B.2.** Post-hoc correlations between N1 sensitivity and arithmetic skills (percentile scores) in T4 | | | | | | | | |
| --- | --- | --- | --- | --- | --- | --- | --- | --- |
| Time point | N1 sensitivity | Hemisphere | Cognitive measure | *n* | Spearman ρ | *p* | *p_FDR_* |  |
|  |  |  |  |  |  |  |  |  |
| T4 | digit-false font | left | HRT addition | **27** | **0.62** | **0.001** | **0.020** |  |
|  |  |  | HRT comparison | *27* | *0.43* | *0.024* | *0.120* |  |
|  |  |  | HRT completion | **27** | **0.56** | **0.002** | **0.020** |  |
|  |  |  | HRT subtraction | *27* | *0.44* | *0.023* | *0.120* |  |
|  |  |  | HRT writing speed | 27 | -0.13 | 0.52 | 0.649 |  |
|  |  |  |  |  |  |  |  |  |
|  |  | right | HRT addition | 27 | 0.08 | 0.708 | 0.745 |  |
|  |  |  | HRT comparison | 27 | 0.05 | 0.822 | 0.822 |  |
|  |  |  | HRT completion | 27 | 0.16 | 0.424 | 0.649 |  |
|  |  |  | HRT subtraction | 27 | 0.15 | 0.47 | 0.649 |  |
|  |  |  | HRT writing speed | 27 | -0.14 | 0.49 | 0.649 |  |
|  |  |  |  |  |  |  |  |  |
|  |  |  |  |  |  |  |  |  |
|  | digit-letter | left | HRT addition | 26 | 0.42 | 0.033 | 0.132 |  |
|  |  |  | HRT comparison | 26 | 0.25 | 0.222 | 0.493 |  |
|  |  |  | HRT completion | 26 | 0.12 | 0.552 | 0.649 |  |
|  |  |  | HRT subtraction | 26 | 0.37 | 0.067 | 0.223 |  |
|  |  |  | HRT writing speed | 26 | -0.18 | 0.386 | 0.649 |  |
|  |  |  |  |  |  |  |  |  |
|  |  | right | HRT addition | 24 | 0.34 | 0.109 | 0.273 |  |
|  |  |  | HRT comparison | 24 | 0.18 | 0.39 | 0.649 |  |
|  |  |  | HRT completion | 24 | 0.11 | 0.61 | 0.678 |  |
|  |  |  | HRT subtraction | 24 | 0.34 | 0.102 | 0.273 |  |
|  |  |  | HRT writing speed | 24 | -0.18 | 0.392 | 0.649 |  |
|  |  |  |  |  |  |  |  |  |
| FDR = False Discovery Rate correction for 20 tests. HRT = Heidelberger Rechentest (arithmetic skills). Bold text indicates results at pFDR < 0.05; Italic text indicates results at p <0.05. | | | | | | | | |

## N1 amplitude association with arithmetic skills (raw scores)

The following analyses were performed separately with left and right hemisphere amplitudes. We examined the association between digit sensitivity at T4 and T5 and arithmetic skills at those time points. The analysis on raw scores at T4 yielded a significant association between N1 digit-false font differences in the left hemisphere and the arithmetic (HRT) total raw score (Spearman ρ = 0.64, *p* = .001, *p_FDR_ =* 0.036; FDR correction for 36 tests). No other association was significant, although there was a trend in T5 for a negative association between right N1 digit-false font sensitivity and HRT arithmetic skills (uncorrected *p* = 0.048; all other *ps* > 0.091). Posthoc analyses of arithmetic subtests at T4, supported this association with coarse N1 digit sensitivity (addition, completion, multiplication and subtraction, *ps_FDR_* < .027, FDR corrected for 20 tests).
